# Auditory stream segregation using bandpass noises: evidence from event-related potentials

**DOI:** 10.3389/fnins.2014.00277

**Published:** 2014-09-12

**Authors:** Yingjiu Nie, Yang Zhang, Peggy B. Nelson

**Affiliations:** ^1^Department of Communication Sciences and Disorders, James Madison UniversityHarrisonburg, VA, USA; ^2^Department of Speech-Language-Hearing Sciences, University of MinnesotaTwin-Cities, MN, USA; ^3^Center for Neurobehavioral Development, University of MinnesotaTwin-Cities, MN, USA

**Keywords:** attention, auditory stream segregation, bandpass noise, MMN, P300, P3b, spectral separation, temporal pattern

## Abstract

The current study measured neural responses to investigate auditory stream segregation of noise stimuli with or without clear spectral contrast. Sequences of alternating A and B noise bursts were presented to elicit stream segregation in normal-hearing listeners. The successive B bursts in each sequence maintained an equal amount of temporal separation with manipulations introduced on the last stimulus. The last B burst was either delayed for 50% of the sequences or not delayed for the other 50%. The A bursts were jittered in between every two adjacent B bursts. To study the effects of spectral separation on streaming, the A and B bursts were further manipulated by using either bandpass-filtered noises widely spaced in center frequency or broadband noises. Event-related potentials (ERPs) to the last B bursts were analyzed to compare the neural responses to the delay vs. no-delay trials in both passive and attentive listening conditions. In the passive listening condition, a trend for a possible late mismatch negativity (MMN) or late discriminative negativity (LDN) response was observed only when the A and B bursts were spectrally separate, suggesting that spectral separation in the A and B burst sequences could be conducive to stream segregation at the pre-attentive level. In the attentive condition, a P300 response was consistently elicited regardless of whether there was spectral separation between the A and B bursts, indicating the facilitative role of voluntary attention in stream segregation. The results suggest that reliable ERP measures can be used as indirect indicators for auditory stream segregation in conditions of weak spectral contrast. These findings have important implications for cochlear implant (CI) studies—as spectral information available through a CI device or simulation is substantially degraded, it may require more attention to achieve stream segregation.

## Introduction

Auditory stream segregation (also referred to as auditory streaming) is an auditory process that occurs naturally in daily life. When listening to a talker in a party or following a melody played by an instrument in an orchestra, listeners with normal hearing interpret the mixture of sounds in such a way that sounds from different sources are allocated to individual sound generators. Research has demonstrated that auditory stream segregation may operate on various physical properties, such as the sound spectrum (Bregman and Campbell, 1971; Warren and Obusek, 1972; van Noorden, 1975; Dannenbring and Bregman, 1976a,b; Bregman et al., 1999) and the temporal envelopes (Singh and Bregman, 1997; Vliegen et al., 1999; Vliegen and Oxenham, 1999; Grimault et al., 2000, 2001, 2002; Roberts et al., 2002). Behavioral (van Noorden, 1975; Botte et al., 1997; Brochard et al., 1999) and neurophysiological (Sussman et al., 1998a, 2005; Sussman and Steinschneider, 2009) studies have further indicated that listeners′ voluntary attention facilitates stream segregation.

Behavioral laboratory studies (e.g., van Noorden, 1975, for a review, see Moore and Gockel, 2002) have indicated that frequency separation and stimulus presentation rate are critical for the formation of auditory streams. The identification of the temporal coherence boundary (TCB) and the fission boundary (FB) (van Noorden, 1975) are amongst the earlier suggestions of ways in which voluntary attention may influence stream segregation. In the van Noorden study, when a frequency separation of two potential tonal streams was larger than the TCB or smaller than the FB, a listener would perceive two streams and one stream, respectively, regardless of their focused attention. When the frequency separation fell in between TCB and FB, offering an ambiguous cue for segregation/integration, a listener could hold either the integrated or the segregated perception depending on directed attention. Brochard et al. (1999) further investigated the role of attention by presenting interleaved subsequences (streams) of tones to normal-hearing listeners. They evaluated attentional effort for stream segregation by measuring the threshold of a temporal offset of a given tone in a focused subsequence (stream) for a listener to detect an irregular rhythm in that subsequence (stream). Their findings showed that more attentional effort was needed for stream segregation when the frequency separation between the subsequences was smaller.

The effect of voluntary attention in auditory stream segregation has also been studied using neurophysiological methods. One important measure was the mismatch negativity (MMN) response. The MMN is typically elicited by automatic change detection in a passive listening oddball paradigm, in which a frequent auditory stimulus (i.e., the standard) is repeatedly presented, while an infrequent stimulus (i.e., the deviant) occasionally replaces the standard (Ford and Hillyard, 1981; Nordby et al., 1988a,b). The MMN is topographically represented by a negative centro-frontal scalp distribution in the temporal window of 100–300 ms post the onset of the change, reflecting pre-attentive detection of the deviant irrespective of attentional efforts (for reviews, see Näätänen, 1995; Näätänen et al., 2007). The MMN can also be elicited during attentive listening (for reviews, see Picton et al., 2000a; Näätänen et al., 2007), but tends to overlap with a negative component N2b that also shows a negative centro-frontal scalp distribution (Näätänen et al., 1982; Novak et al., 1990, for a review, see Folstein and Van Petten, 2008). Previous studies have demonstrated that the MMN can be measured as an indirect index of stream segregation in passive listening when the concurrent auditory streams are sufficiently different in frequency (Sussman et al., 1998a, 1999, 2001; Winkler et al., 2001, 2003; Yabe et al., 2001). For example, Sussman et al. (1999) presented normal-hearing listeners with standard stimulus sequences comprising two interleaved subsequences (streams) of tones. Deviants were inserted into either subsequence. In the unattended condition, MMNs to the deviants were elicited only when the tones were presented at a fast rate wherein stream segregation was induced. Studies have further demonstrated that the MMN measure could reflect the facilitative role of voluntary attention in stream segregation (Sussman et al., 1998a, 2005).

Another important neurophysiological measure to examine the role of voluntary attention in stream segregation is the P300 response, which is known to index attentional shift to novelty detection (Sutton et al., 1965). While P3a (an earlier component of the P300 family with a frontal distribution) reflects obligatory processes (e.g., involuntary attention shift) in the passive or attentive listening condition, the P3b with a posterior parietal distribution is associated with voluntary attentional orientation to the deviants in the attentive listening condition (Knight and Nakada, 1998; Knight and Scabini, 1998; Corbetta and Shulman, 2002), or with classifying initially uncategorized events into a discrete group (Friedman et al., 2001). Sussman and Steinschneider (2009) studied how two sets of tones with various frequency separations were segregated into different auditory streams or integrated into one stream in adults and children. They assessed P3b in the attentive condition and found that in adults, higher P3b amplitudes were associated with larger inter-stream frequency separation, which presumably led to better performance in stream segregation.

Overall, the published ERP data have shown that the MMN and P300 measures can reflect segregation success or failure along with behavioral tests. In cases of children with hearing loss, and especially children with cochlear implants (CIs), ERP measures of stream segregation could serve as indirect indicators of signal-processing or rehabilitative program success. However, previous ERP studies have only used tonal stimuli with clear spectral separation between the auditory streams. The current investigation extended this line of research by examining whether the MMN and P300 measures could be reliably elicited for auditory stream segregation based on noise stimuli with or without clear spectral contrast. The revelation of normal-hearing listeners′ ability to pre-attentively segregate the two noise streams may offer a baseline against which future research on CI users can be compared.

Behavioral studies on normal-hearing adult listeners showed that bandpass-filtered noises with separated (Dannenbring and Bregman, 1976b) and overlapped spectra (Bregman et al., 1999) could be perceived as from different auditory streams. The bandpass-filtered noise stimuli may simulate the stimulations with less salient spectral differences through a CI electrode array, whose users only demonstrate 8–10 effective auditory filters (Fu et al., 1998; Friesen et al., 2001; Nelson et al., 2003). However, it remains inconclusive how CI users perform stream segregation based on input with degraded spectral information (Chatterjee et al., 2006; Hong and Turner, 2006; Cooper and Roberts, 2009). While Hong and Turner (2006) showed CI users could segregate streams of pure tones at different frequencies, Cooper and Roberts (2009) argued that they were unable to segregate streams based on electrode-separation which represents spectral separation in the electrical stimulation. Chatterjee et al. (2006) noted that only one out of five CI subjects showed definitive electrode-separation based stream segregation. Most recently, the work of Böckmann-Barthel et al. (2014) suggested that CI users were able to segregate different streams of tones adequately differing in frequency with a processing time course comparable to normal-hearing listeners. Studying how the reduced spectral separation operates for stream segregation at various processing stages (i.e., pre-attentive and attentive) in normal-hearing listeners may provide a foundation to better understand stream segregation based on spectral separation in CI users.

One of the speech cues that is well preserved for CI users is rhythm (e.g., McDermott, 2004). A recent behavioral study (Micheyl and Oxenham, 2010) has indicated that the steady rhythm of a tone series can be a cue for listeners to segregate the tone series from another. In that study, reiterated triplets of tones were presented in an ABA pattern with A tones quasi-randomly occurring temporally and B tones occurring at a fixed B-to-B interval. The listeners were able to identify the temporal displacement of the last B tone to some extent even when A and B tones were set to the same frequency. Thus, detecting the deviant B-to-B interval at the end of a sequence would require the perception of the organized B tones into one stream on the basis of the built-in temporal pattern.

The current study followed Micheyl and Oxenham (2010) in the use of a deviant in a rhythmic stream to assess the stream segregation process. Unlike the previous study, sequences of two interleaved subsequences of noise bursts, namely A and B bursts, were presented. While the temporal position of each A burst was quasi-randomized between the two adjacent B bursts, the B bursts were presented steadily (i.e., with a constant B-to-B onset-to-onset interval) except that the last B burst was delayed (i.e., presented with a longer B-to-B onset-to-onset interval), in half of the stimulus sequences. It was anticipated that, if the steadily presented B bursts were perceived as one stream distinct from the stream comprising the unsteadily-presented A bursts, a delayed B burst would be perceived as a deviant breaking the steady rhythm of the B-to-B gaps. In contrast, subjects may have also integrated the A and B bursts as one stream consisting of arrhythmic elements. The jittered timing of A bursts would introduce uncertainty to an A-to-B gap, making an A-to-B gap an ineffective cue to detect a delayed B burst. Therefore, the detection of the deviant in the rhythmic stream can be used as an indirect indicator for stream segregation.

The goal of the current study was to examine neural correlates of stream segregation for stimuli with weak spectral information in passive listening as well as in attentive listening conditions. We were specifically interested in addressing three questions. First, would the noise-based stimulus paradigm yield measurable neurophysiological responses to the delay of the last B bursts to indirectly index stream segregation? Second, is the selected spectral separation between two sets of bandpass-filtered noise, simulating a moderate electrode-separation through a CI processor, necessary to show differences in stream segregation at the pre-attentive level? Third, does voluntary attention facilitate stream segregation for the noise stimuli? The MMN responses in a passive listening condition were measured to indirectly index pre-attentive stream segregation, and the P3b responses in an attentive listening condition were measured to indirectly index stream segregation with involvement of voluntary attention.

## Materials and methods

### Subjects

Nine right-handed adult listeners (5 females and 4 males; 19–37 years old) participated in the study. Their hearing thresholds were no greater than 20 dB HL at audiometric frequencies of 250, 500, 1000, 1500, 2000, 3000, 4000, 6000, and 8000 Hz on the right side. The research procedure was approved by the Institutional Review Board at the University of Minnesota for experiments with human subjects. Each subject gave written informed consent.

### Stimuli and test procedure

**Figure 1:**
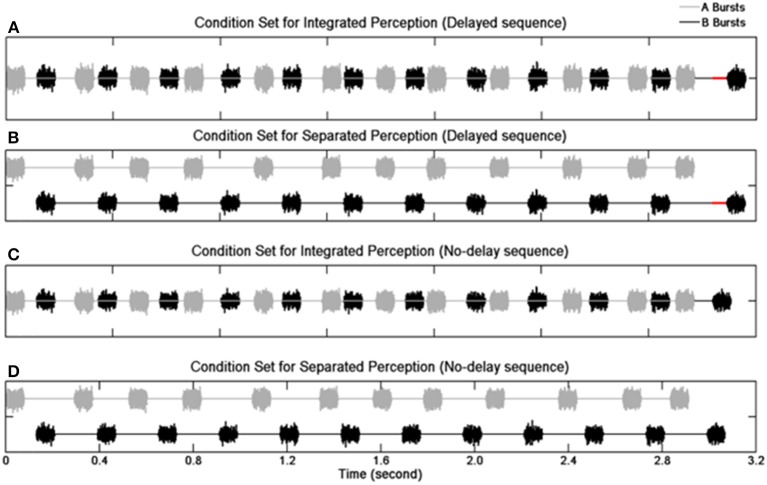
**Schematic illustration of the stimulus paradigm**. Waveforms of noise bursts are plotted. Panels **(A,B)** show the delayed sequences with the red solid lines indicating the duration of the delay for the 12th B burst. Panels **(C,D)** show the no-delay sequences. Panels **(A,C)** depict the condition when individual sequence elements result in an integrated perception, and panels **(B,D)** depict the condition when individual elements result in a segregated perception.

Sequences of 12 pairs of A and B noise bursts were presented (Figure 1). The duration of an A or B burst was 80 ms including 8-ms rise/fall time. The B burst sequences maintained an onset-to-onset interval of 340 ms except for the last (12th) B burst whose onset was either delayed or not delayed. In the delayed sequences, the 12th B bursts were delayed from their nominal temporal positions by 30 ms. In the no-delay sequences, the 12th B bursts were presented at the nominal temporal location. The total duration was 3.1 s for the delayed sequences and 3.07 s for the no-delay sequences. The A bursts (expect the first one) were pseudo-randomly placed between two successive B bursts. The stimulus-onset asynchrony (SOA)—defined as an interval between the onsets of two consecutive bursts (i.e., the onsets of an A burst and its adjacent B bursts, or the onsets of a B burst and its adjacent A bursts)—was assigned with a nominal mean value of 130 ms. The real SOAs varied between 90 and 170 ms due to the jittering of the A bursts. Specifically, the onset of any given non-initial A burst was advanced or delayed by an amount of time ranging from 0 to 40 ms. The amount of jitter was determined based on a pilot study.

Manipulation of spectral separation between the burst sequences used two types of noises, broadband noise and band-pass filtered noise. For the broadband noise stimuli with no spectral separation (no-SSEP), an independent Gaussian noise was created for each element of a given stimulus sequence for the A and B burst sequences. To introduce spectral separation (SSEP), the A bursts were generated with a 4th-order Butterworth filter (cutoff frequencies at 2149 and 7000 Hz), and a bandpass filter between 200 and 1426 Hz was utilized to generate the B bursts. The slope of the filters was set at 12 dB/octave to resemble the shallow filter slope in CI users (Anderson et al., 2011). Altogether, the bandpass filtering procedure resulted in a 2.86-octave distance between the center frequencies of the A and B bursts with a 0.59-octave distance between the lower edge frequency of A bursts and the higher edge frequency of the B bursts.

Listeners were seated comfortably in a chair in an acoustically-attenuated and electrically-isolated chamber (ETS-Lindgren Acoustic Systems). Stimulus presentation used the EEvoke software (ANT Inc., The Netherlands). The sounds were presented through an insert earphone (Etymotic Research ER-3A) monaurally to the right ear. The sound level was set at 60 dB above the subject′s hearing threshold for a 1000 Hz sine wave tone.

Both passive listening and attentive listening conditions were administered for the two types of stimulus setup (no-SSEP and SSEP), which resulted in four stimulus blocks lasting approximately 2 hours. All subjects were presented the same stimuli; the order of the stimulus blocks was counterbalanced across the subjects. The subjects repeated the same four stimulus blocks on a different day to allow sufficient number of trials for ERP data analysis. In the passive listening condition, subjects were directed to ignore the acoustic stimuli while watching a muted movie with subtitles. In the attentive listening condition, the subjects were instructed to respond only to the delayed sequences by pushing a key on a computer keyboard. Each stimulus block contained 120 independently generated stimulus sequences with 60 delayed and 60 no-delay sequences arranged in a random order. The offset-to-onset inter-stimulus-sequence interval was randomized between 900 and 1000 ms. The inter-block break interval was 5–10 min.

Prior to the ERP experiments, all subjects had participated in a behavioral experiment with the same stimulus paradigm and more spectral separations between A and B bursts for 10 days spread over 1–2 months for a total of approximately 15 hours of listening task to detect the delayed 12th B bursts^1^.

### EEG recording and analysis

Continuous EEG was recorded (bandwidth = 0.016–200 Hz; sampling rate = 512 Hz) using the ASA-Lab system with a REFA-72 amplifier (TMS International BV) and a 64-channel WaveGuard cap (ANT Inc., The Netherlands). The ground was positioned at AFz, and the default reference for the REFA-72 amplifier was the common average of all connected unipolar electrode inputs. Impedances of individual electrodes were at or below 5 kΩ.

ERP averaging was performed offline in BESA (Version 6.0, MEGIS Software GmbH, Germany) following recommended guidelines (Picton et al., 2000b; DeBoer et al., 2007). The automated EOG correction algorithm in BESA was applied to the EEG data with the threshold parameters at 150 μV for HEOG and 250 μV for VEOG. The ERP epoch length was 700 ms, including a pre-stimulus baseline of 100 ms. The ERP data were bandpass filtered at 0.53–40 Hz and re-referenced to the average of the recordings at the mastoid channels. Trials with potentials exceeding ±50 μV were rejected. For each subject, the ERP waveforms recorded in the two separate sessions for the same condition were first analyzed individually and then combined with weighted averaging in BESA. Unweighted averaging was calculated for the grand mean at the subject group level.

The standard stimuli were the ending (i.e., 12th) B bursts of the no-delay sequences, and the deviant stimuli were those ending B bursts of the delayed sequences. Difference ERP waves were obtained by subtracting the standard ERPs from the deviant ERPs in each of the four conditions (i.e., no-SSEP passive listening, SSEP passive listening, no-SSEP attentive listening, and SSEP attentive listening). In the passive listening condition, the individual subjects had the total number of accepted trials in the range of 101–118 for either the delayed or no-delay stimulus sequences. In the attentive listening condition, the trials with a behaviorally incorrect response were excluded from the ERP analysis; the total number of correct responses was in the range of 59–114 for the SSEP stimuli, and the correct responses were in the range of 39–101 for the no-SSEP stimuli. The counts of false alarms were 0–19 for the SSEP stimuli and 4–46 for the no-SSEP stimuli. Given the insufficient number of trials, no ERP analysis was performed on the false-alarm trials for the no-delay 12th B bursts.

Global field power (GFP) was calculated by computing the standard deviation of the amplitude data across the 64 electrodes at each sampling point as an unbiased estimate independent of electrode selection (Lehmann and Skrandies, 1980; Hamburger and Burgt, 1991). The time windows for ERP component analysis were selected based on the grand-mean GFP plots and scalp topography maps. The ERP analysis windows were confirmed by visual inspection of the grand-mean ERP waveform overlay plots using single channels. Temporal windows of 200, 50, and 50 ms around the GFP peaks for P3b, MMN, and MMN/N2b responses were chosen, respectively. Specifically, for the attentive listening conditions, the windows were 244–444 ms for the SSEP stimuli and 300–500 ms for the no-SSEP stimuli to assess the P3b component, respectively. For the passive listening conditions, the window of 280–330 ms was selected for both SSEP and no-SSEP stimuli. To assess the MMN/N2b component in attentive listening, the windows were 97–147 ms for the SSEP stimuli and 174–224 ms for the no-SSEP stimuli. The CPz electrode was used for P3b analysis, and the Fz was used for MMN analysis. Given the small amplitude of the N2b response, six centro-frontal channels (FC1, FC2, FCz, C1, C2, and Cz) were grouped in our analysis. For each subject, mean amplitude values in the selected time windows for the ERP components were calculated for the deviant-to-standard difference waveforms under the four conditions (i.e., two spectral contrasts by two attentional conditions) (Luck, 2005; Clayson et al., 2013).

One-tailed one-sample *t*-tests were conducted on these average amplitudes independently under each of the four conditions. The criterion for statistical significance was divided by six (i.e., α = 0.0083) to correct for the number (i.e., six) of comparisons. A paired two-tailed *t*-test was carried out to compare the average amplitudes obtained for the SSEP stimuli against those obtained for the no-SSEP stimuli for the attentive listening condition.

To assess the strength of the ERP activities relative to the baseline independent of electrode selection, *z*-scores were calculated with Bonferroni correction for the post-stimulus GFP at each sampling point (Rao et al., 2010; Zhang et al., 2011). Sustained latency intervals of at least 20 ms or longer (Rao et al., 2010; Zhang et al., 2011) were highlighted where *z*-scores indicated the GFP was significant.

### Analysis on behavioral data during attentive listening

For each subject, the hit rate for the delayed sequences and the false alarm rate for the no-delay sequences were combined across the two recording sessions for the no-SSEP and SSEP stimuli. The behavioral scores were then converted to *d*′ scores (Macmillan and Creelman, 2005). A paired two-tailed *t*-test was performed to compare the *d*′ scores between the SSEP and no-SSEP stimulus conditions.

### Correlational analysis for P3b response and behavioral performance

A *post-hoc* Pearson correlation was conducted for the P3b measure (separate tests for amplitude and latency) and the behavioral *d*′ measure in the attentive listening condition. Considering the small number of subjects in our study, we pooled the SSEP and no-SSEP stimulus conditions together.

## Results

### P3b in the attentive condition

**Figure 2:**
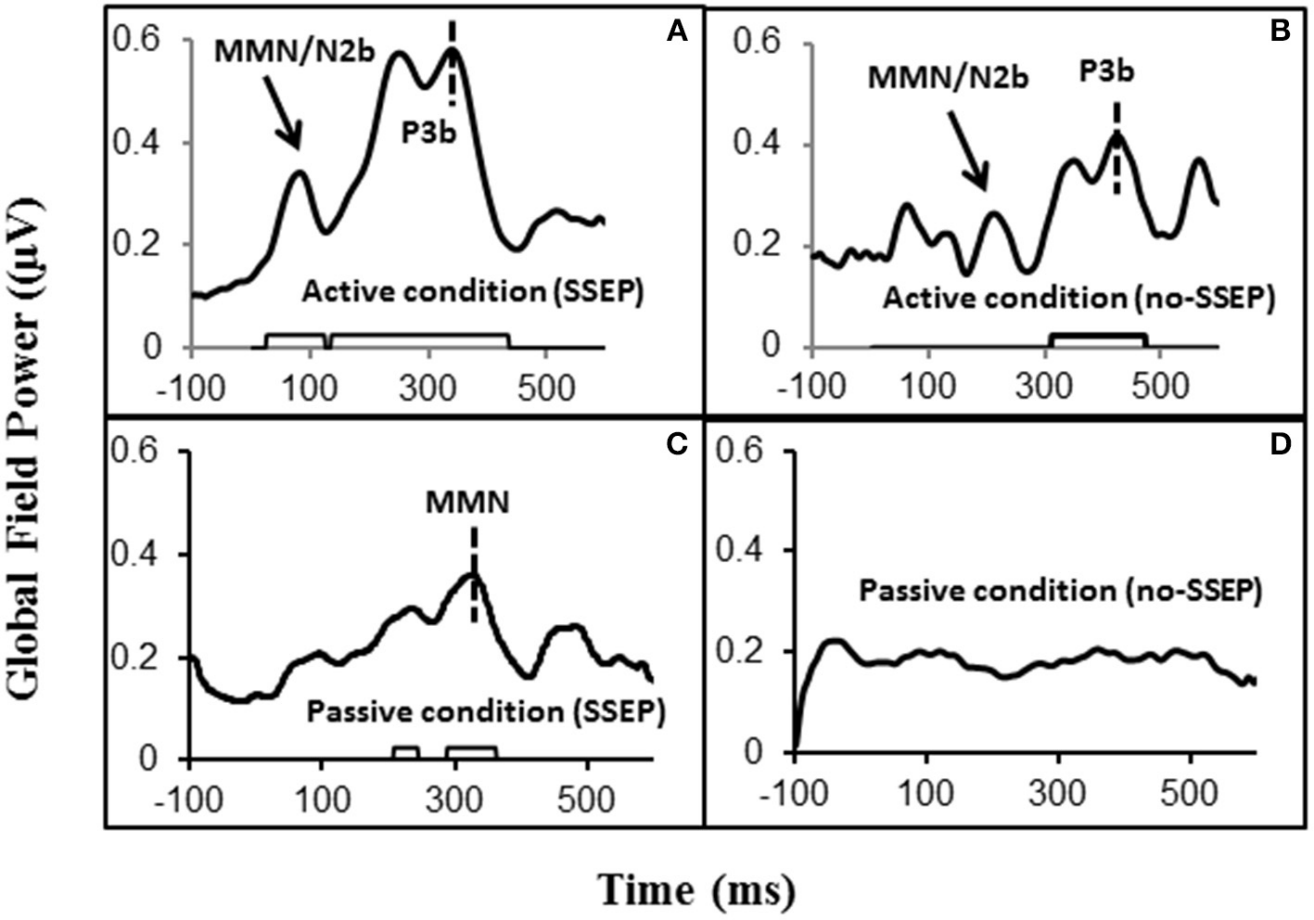
**Global field power (GFP) data obtained from the grand-mean ERP deviant-to-standard difference waveforms**. Panels **(A,B)** respectively show GFPs for the SSEP and no-SSEP stimuli during attentive listening. Panels **(C,D)** show GFPs for the SSEP and no-SSEP stimuli during passive listening. The bars along the x-axes in **(A–C)** show significant activities as determined from *z*-scores relative to the 100-ms pre-stimulus baseline. Panel **(D)** shows an absence of significant GFP activities. The dashed vertical lines in **(A,B)** mark the GFP peaks of P3b. The dashed vertical line in **(C)** indicates the GFP peak of MMN for the SSEP stimuli. The GFP peaks falling in the MMN/N2b time window are indicated for the SSEP **(A)** and no-SSEP stimuli **(B)** by the arrows.

**Figure 3:**
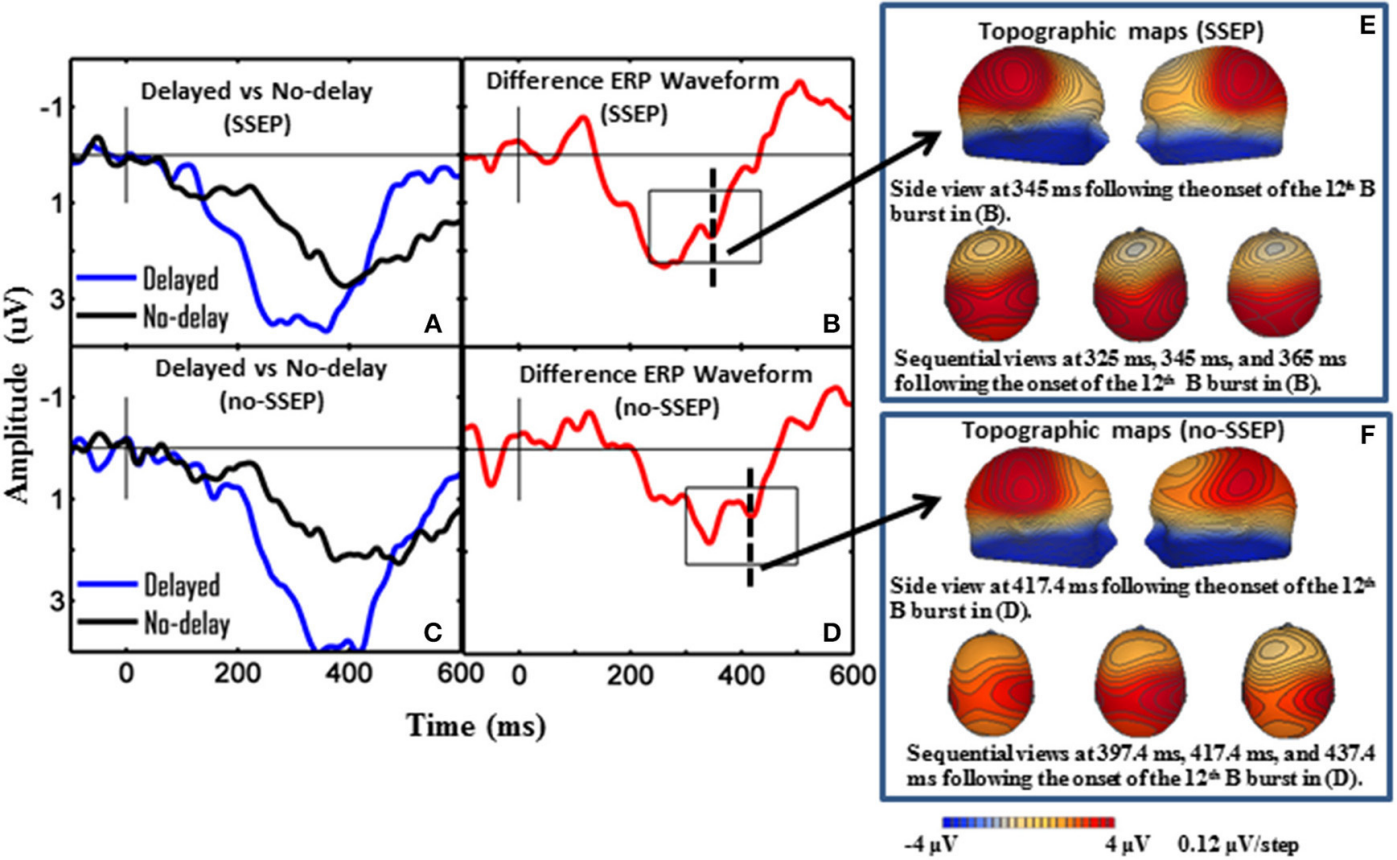
**Grand-mean ERP waveforms and grand-mean ERP deviant-to-standard difference waveforms from CPz**. Panels **(A–D)**, respectively show attentive listening for the SSEP stimuli, attentive listening for the no-SSEP stimuli, passive listening for the SSEP stimuli, and passive listening for the no-SSEP stimuli. The topographic maps for the P3b peaks are shown in **(E)** (SSEP stimuli) and panel **(F)** (no-SSEP stimuli). The solid vertical lines depict the onsets of the 12th B bursts. The thick dashed vertical lines in **(B,D)** mark the P3b peaks. The boxes surrounding the peaks in **(B,D)** show the analysis windows for the P3b activity.

Significant P3b responses were observed for both the SSEP and no-SSEP stimuli in the attentive listening condition (Table 1 and Figure 2). Grand-mean ERP waveforms at the CPz channel and topographic maps showed a strong positive posterior parietal distribution, which peaked earlier for the SSEP stimuli (at 345 ms) than for the no-SSEP stimuli (at 417 ms) (Figure 3). But there was no significant difference in the P3b amplitude between the SSEP and no-SSEP stimulus conditions [*t*_(1, 8)_ = 0.89, *p* = 0.395].

**Table 1:** **Mean amplitudes of the ERP components calculated from the difference waveforms (i.e., delayed minus no-delay) for the 12th B bursts**.

	**Attentive condition (P3b)**		**Attentive condition (MMN/N2b)**		**Passive condition (MMN)**	
	**SSEP**	**no-SSEP**	**SSEP**	**no-SSEP**	**SSEP**	**no-SSEP**
Average amplitude of difference waveforms (μV)	1.70 (0.90)	1.25 (1.45)	−0.44 (1.30)	−0.16 (1.25)	−0.85 (1.60)	0.45 (1.41)
*t-value* (one-tailed one-sample *t*-test for the difference waveforms)	*t*_(1, 8)_ = 5.34	*t*_(1, 8)_ = 2.93	*t*_(1, 8)_ = −1.01	*t*_(1, 8)_ = −0.39	*t*_(1, 8)_ = −1.59	*t*_(1, 8)_ = 0.89
*p-value* (statistical significance level α < 0.0083)	0.0003	0.0022	0.1707	0.3534	0.0749	0.2005

*Standard deviations are shown in the parentheses. The t- and p-values were calculated from one-tailed one-sample t-tests comparing the amplitudes of the ERP component with the zero baseline*.

**Figure 4:**
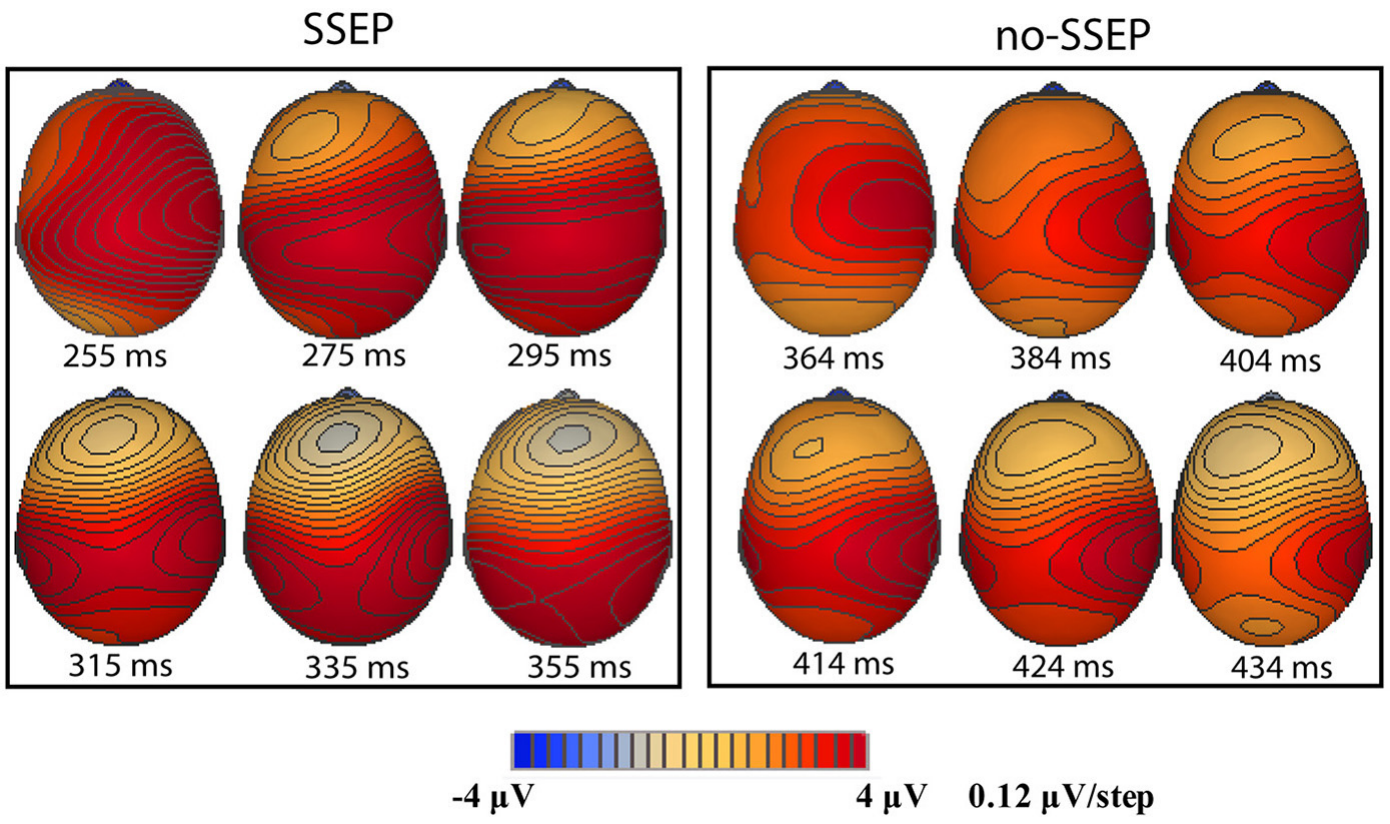
**Sequential topographic maps illustrating a frontal-to-parietal shift in the positive potential maximum at selected time points post the onset of the 12th B bursts for the SSEP (left panel) and no-SSEP (right panel) stimuli, respectively**.

Sequential topographic maps exhibited a positive potential maximum moving from the frontal area to the posterior parietal area in the time window starting from around 255 ms post stimulus to around 355 ms for the SSEP stimuli, and from 364 to 434 ms for the no-SSEP stimuli (Figure 4). The earlier frontal positive distributions indicated a possible occurrence of the P3a component before the occurrence of the posterior-parietal P3b for both SSEP and no-SSEP stimuli.

### MMN/N2b component in the attentive condition

**Figure 5:**
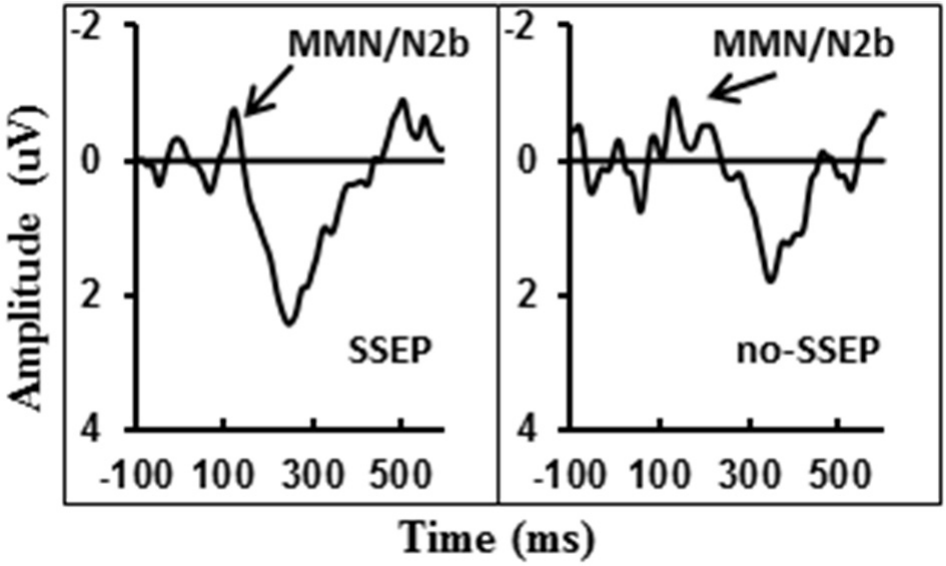
**Grand-mean ERP deviant-to-standard difference waveforms grouped across six centro-frontal channels (FC1, FC2, FCz, C1, C2, and Cz) in attentive listening for SSEP (left panel) and no-SSEP (right panel) stimuli**. The MMN/N2b-like peaks are indicated by the arrows.

Visual inspection of the grand-mean ERP difference waveforms (Figure 5) indicated the presence of a small MMN/N2b component at the centro-frontal sites preceding the P3b response during attentive listening. However, this N2b component was not significantly different from the zero baseline in either the SSEP or the no-SSEP stimulus condition (Table 1).

### MMN in the passive condition

**Figure 6:**
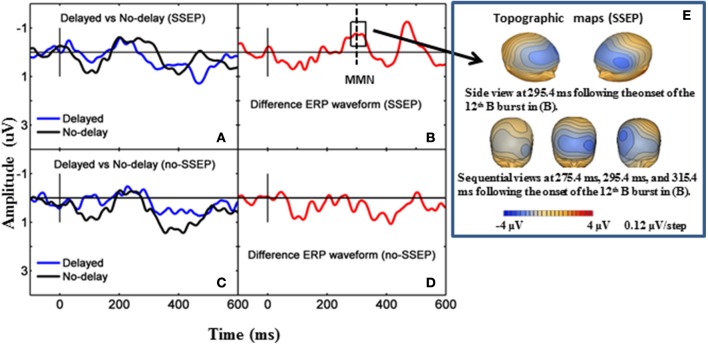
**Grand-mean ERP waveforms and grand-mean ERP deviant-to-standard difference waveforms from the Fz electrode**. Panels **(A,B)** stand for passive listening in the SSEP stimulus condition, and panels **(C,D)** stand for passive listening in the no-SSEP stimulus condition. The topographic maps for the MMN peaks are shown in **(E)** for the SSEP stimuli. The solid vertical lines mark the onsets of the 12th bursts. The dashed vertical line in **(B)** mark the MMN peak. The box around the peak in **(B)** represents the analysis window for the MMN.

Both GFP data and Fz data showed no presence of MMN during passive listening when there was no spectral separation between the A and B burst sequences (i.e., no-SSEP stimulus condition) (Figures 2 and 6). In the SSEP stimulus condition, however, a discrepancy was observed between the GFP waveform analysis and the Fz electrode waveform analysis on the MMN data. The GFP data showed significant MMN activities in the time windows of 209–241 and 290–362 ms for the SSEP stimuli during passive listening (Figure 2). Based on the GFP data, the MMN peak latency in the SSEP stimulus condition was found to be at 315 ms. In contrast, the MMN amplitudes at the Fz channel failed to reach statistical significance for the SSEP stimuli (Figure 6). Inspection of the individual data showed that one subject had a mismatch positivity response at Fz, which could have contributed to the discrepancy between the GFP and MMN statistical results.

### Behavioral data

Subjects showed greater *d*′ values for the SSEP stimuli than for the no-SSEP stimuli [*t*_(1, 8)_ = 5.18, *p* < 0.001] (Table 2). Thus, the presence of spectral contrast facilitated the detection of the delayed 12th B bursts in the SSEP stimulus condition.

**Table 2:** **Hit rates, false alarm rates, and *d*′-values calculated from subjects' behavioral responses when attempting to identify the delayed 12th B bursts during attentive listening**.

	**SSEP**		**no-SSEP**	
	**Range**	**Average (SD)**	**Range**	**Average (SD)**
Hit rate (%)	49–95	74 (15)	32–84	65 (15)
False alarm rate (%)	0–16	5 (5)	3–38	17 (11)
*d*′	1.46–3.33	2.56 (0.62)	0.57–2.29	1.45 (0.58)

### Brain-behavioral correlation during attentive listening

**Figure 7:**
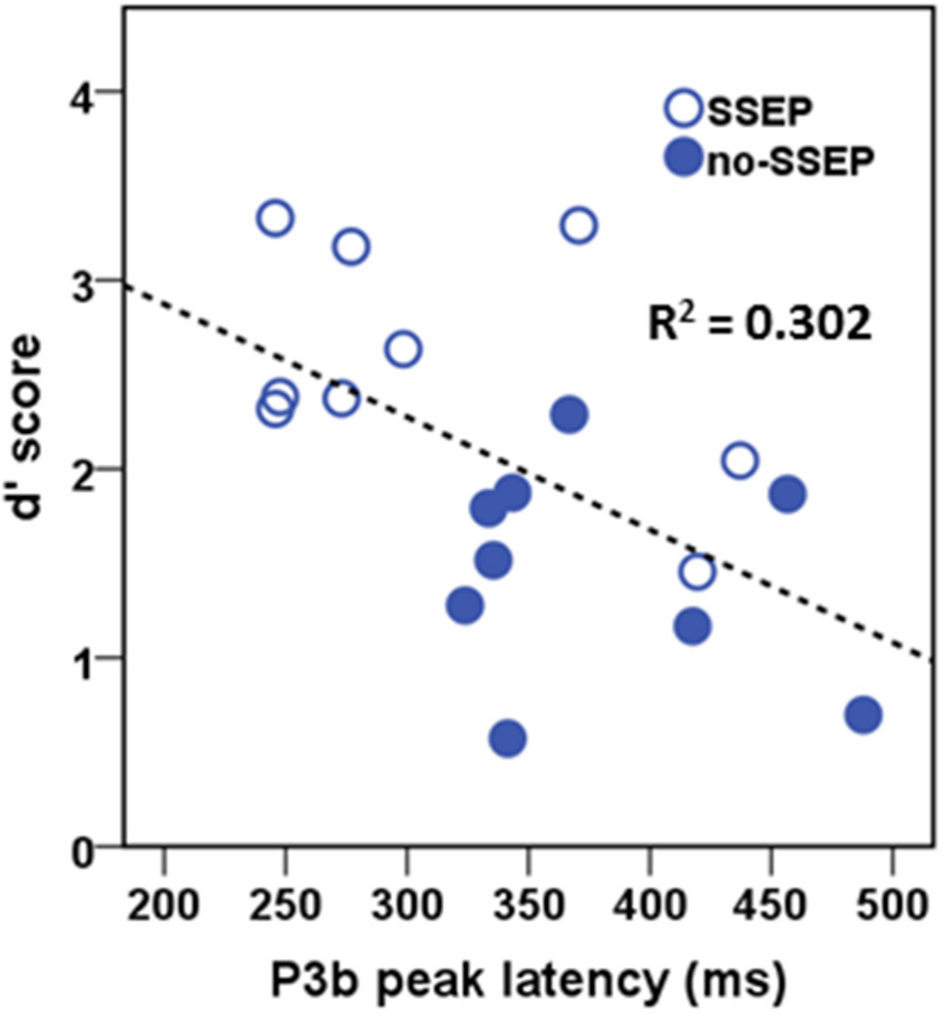
**Correlational scatterplot illustrating the relationship between behavioral *d*′ score and the P3b peak latency in attentive listening**. The data were pooled across the SSEP and no-SSEP stimulus conditions.

In the attentive listening condition, a significant correlation was found between the P3b latency values and the *d*′ scores (Figure 7). Higher *d*′ scores for detecting the change in the final position of the B sequences were associated with earlier P3b responses (*r* = −0.549, *p* < 0.05). However, the *d*′ scores were not significantly correlated with the P3b amplitude data.

## Discussion

Overall, results in both attentive and passive listening conditions confirm that ERP measures may be reliable indirect indicators of stream segregation based on less distinctive spectral separation cues. In the passive condition, significant MMN activities were observed in the GFP data for the SSEP stimuli but not for the no-SSEP stimuli, indicating that spectral separation between A and B bursts was necessary for stream segregation at the pre-attentive level. In the attentive condition, spectral separation between the A and B bursts contributed to better performance in stream segregation. Conscious perception of stream segregation was indirectly indicated by the presence of significant P3b activities for both SSEP and no-SSEP stimuli. Moreover, better behavioral performance was correlated with earlier P3b peak response.

### Stimulus paradigm and effect of rhythm on stream segregation

The first goal of the current study was to investigate whether the stimulus paradigm used in our behavioral study (Nie and Nelson, 2010) could elicit reliable ERP measures to indirectly index stream segregation with noise bursts. Similar paradigms with tonal stimuli have been used in behavioral psychophysical studies on stream segregation (e.g., Roberts et al., 2002; Hong and Turner, 2006; Micheyl and Oxenham, 2010). Our behavioral data showed feasibility of this paradigm with noise stimuli, and our ERP data here further demonstrated that a clear P3b response and a late MMN response could be respectively, elicited for the attentive and passive listening conditions in the perceptual/cognitive processing of the temporally displaced element using such a paradigm. It should be noted that the ERP measures (i.e., MMN, P3b, MMN/N2b, and P3a components) reported here are indirect indicators for stream segregation that are specific to the stimulus paradigm. The presence of the ERP components signifies the pre-attentive or conscious detection of the delayed 12th B bursts, which is facilitated by segregation of the A and B sequences based on temporal pattern, spectral separation as well as voluntary attention. We speculate that the stimulus paradigm involving noise bursts (as opposed to tonal stimuli) and rhythmic B sequences intermixed with arrhythmic A sequences may have jointly affected the latency of the MMN responses in individual subjects and increased the inter-subject variability during passive listening.

Our ERP results reveal that the rhythm cue itself is not adequate to generate stream segregation pre-attentively; however, when listeners focus attention on following the rhythm, they may be able to utilize this cue to form stream segregation. In the no-SSEP stimulus condition, the sole cue available for listeners to segregate the streams of A and B bursts was the steady rhythm in the B stream. The presence of P3b for the both SSEP and no-SSEP stimuli suggests that the steady rhythm of B bursts can contribute to stream segregation in addition to spectral separation. This is consistent with the weak segregation effect reported by Micheyl and Oxenham (2010) for ABA tone sequences that differed in rhythm but not in frequency.

Together, the results suggest that the current stimulus paradigm can be a potential tool for future ERP studies on stream segregation in normal as well as clinical populations with degraded auditory signal. We would like to stress that the benefit of this paradigm may be limited by the substantial amount of time (approximately 15 hours) spent on the training/behavioral experiment prior to the ERP study. Further study of the effects of training on ERP results may help address this limitation.

### Effect of spectral separation on stream segregation with bandpass noise stimuli

The second goal of the study was to investigate whether weak (noisy) spectral separation cues using bandpass noises in the current design could generate pre-attentive stream segregation. A series of studies using tonal stimuli (e.g., Sussman et al., 1998b, 1999, 2005) have systematically demonstrated that MMN could be used as an indirect index for stream segregation providing the stimulus paradigm is suitably designed.

The absence of MMN in the no-SSEP stimulus condition in our study indicated the need for spectral separation in addition to the temporal rhythmic cue for the auditory streaming of the noise sequences at the pre-attentive level. In the SSEP stimulus condition, the presence of MMN in response to the delayed 12th B bursts may not have been a result of integrating A and B bursts into one stream. If the A and B bursts were integrated, a stronger integration would be anticipated for the no-SSEP stimuli, which could presumably lead to a stronger MMN to deviants for the no-SSEP stimuli than for the SSEP stimuli. The current findings are contrary to this prediction. Therefore, the MMN elicitation in the delayed sequences would depend on the pre-attentive detection of rhythmic disruption in the B sequences (the deviant B-to-B gaps in contrast to the standard B-to-B gaps stored in the auditory memory), which was aided by the presence of spectral separation between the A and B sequences.

The elicitation of the late MMN activity for the SSEP stimuli suggests that the listeners were able to extract the temporal patterns in the B sequence from the intermixed A-B series. With an SOA of 130 ms, the amount of frequency separation between the two alternating bandpass noises can be allocated into segregated streams at the pre-attentive auditory processing stage. Specifically, the center frequencies of the A and B bursts in the SSEP stimulus condition were separated by 2.86 octaves. This amount of frequency separation would be an unambiguous cue to generate stream segregation for tonal stimuli with a SOA between 90 and 130 ms (van Noorden, 1975; Sussman et al., 1999, 2005). However, the frequency separation between the two sets of bandpass noises was effectively reduced as a result of the 0.59-octave difference between the edge frequencies of the A and B bursts in addition to the use of a shallow filtering slope of 12 dB/octave. Even with the degraded frequency separation of the bandpass-filtered stimuli, there was evidence for pre-attentive stream segregation in the current paradigm.

### Effect of voluntary attention on stream segregation

The third goal was to examine the effect of voluntary attention on stream segregation. Due to different ERP components analyzed for the passive and attentive listening conditions, no statistical comparisons were performed between the ERP measures obtained in the two listening conditions in the current study. Nevertheless, consistent with previous studies (Sussman et al., 1998a, 2005), voluntary attention has been shown to facilitate stream segregation as P3b was consistently elicited for both SSEP and no-SSEP stimuli during attentive listening in our study. In particular, the absence of MMN during passive listening and the presence of P3b during attentive listening for the no-SSEP stimuli clearly demonstrate that when there is no spectral separation between the A and B noise sequences, attentive listening is necessary to allow stream segregation. Despite the lack of spectral cues, voluntarily directing attention toward the built-in temporal patterns allowed listeners to segregate the two auditory streams.

The P3b response has been demonstrated to reflect uncertainty resolution involving the integrative processing of stimulus evaluation and response execution (Verleger, 1997, 2010; Dien et al., 2004). Previous research has shown that shorter P3b latencies and larger P3b amplitudes tend to be associated with greater stimulus salience (e.g., Sussman and Steinschneider, 2009). Despite a strong effect of spectral separation (SSEP vs. no-SSEP) in the behavioral data, our P3b amplitude data did not show significant differences between the SSEP and no-SSEP stimulus conditions. Neither was the P3b amplitude correlated with behavioral accuracy. Thus, the P3b amplitude might not be a proper measure to assess the strength of stream segregation with the current experimental design. In contrast, the P3b latency measure was found to be significantly correlated with behavioral performance in the *post-hoc* analysis. Spectral separation allows increased certainty accompanied by greater behavioral accuracy in evaluating the B sequences with either delay or no delay in the last noise burst. As our study did not incorporate experimental manipulations to differentiate contributions of stimulus evaluation and response selection to P3b activity, further studies would be necessary to explore how P3b amplitude and latency may be related to behavioral accuracy and response times under different processing strategies (e.g., requiring listeners to prioritize response accuracy over speed or vice versa).

### Technical considerations on discrepancies in MMN results

The GFP data and ERP waveform data (at Fz) did not yield consistent results in the MMN analysis for the SSEP stimuli during passive listening. As GFP calculation uses all electrodes and ERP waveform analysis uses only selected electrodes, some minor differences are not unexpected (Miller and Zhang, 2014). The GFP data in our study clearly indicated the occurrence of significant neural activities within the typical MMN time window (100–300 ms post-stimulus) followed by a larger late response. The topographic maps further indicated a frontal distribution with a negative polarity, and the peak MMN activity as shown in GFP occurred at 315 ms after the onset of the delayed 12th B burst (Figure 2C), which was equivalent to 345 ms after the expected onset of a no-delay 12th B burst. Our GFP data are consistent with previous MMN studies on the extraction of complex tone patterns or abstract regularities/rules, which also have reported a similar response pattern with the regular MMN followed by an additional late MMN (Korpilahti et al., 1995; Zachau et al., 2005). Late MMN responses have also been reported in adult subjects for studies involving linguistic stimuli (Hill et al., 2004; Zhang et al., 2005, 2009; Takegata et al., 2008). This late negativity is sometimes referred to as late discriminative negativity (LDN), which has been found mainly in MMN studies on children using linguistic stimuli (Ceponiene et al., 1998, 2003, 2005; Korpilahti et al., 2001; Maurer et al., 2003). Some researchers suggest that the late MMN activity may be an index of attentional reorienting or auditory learning and development (Putkinen et al., 2012) and that it diminishes in the course of child development (Ceponiene et al., 2003).

Unlike the GFP results, the waveform data analysis at the Fz site failed to show the elicitation of significant MMNs at the subject group level. Inspection of the individual data indicated that one subject showed a positive mismatch response (p-MMR). The p-MMR data point was found to be a statistical outlier in terms of its *z*-score, and its inclusion in the group level analysis affected the statistical outcome. The existence of the p-MMR data point only affected the waveform analysis but not the GFP analysis because GFP calculation relies on the absolute amount of deviation irrespective of polarity. The p-MMR responses in the passive listening oddball paradigm have been reported in a number of child studies (e.g., Maurer et al., 2003; Shafer et al., 2010). As there has been no systematic report about adult p-MMR, it is unclear what might have caused the occurrence of one aberrant p-MMR data point in our study. We suspect that it could be related to attentional processing in this individual subject.

### Implications for cochlear implant users

Results from the current study have important implications for the investigation of stream segregation in CI users. The spectral separation used in our SSEP stimuli corresponds to the frequency regions of the lower four channels and the upper three channels of a simulated 8-channel CI (Fu and Nogaki, 2005). With such a large frequency difference, stimulation through CIs may lead to pre-attentive stream segregation. Furthermore, when the frequency cue is limited, temporal rhythm may be an important cue for CI users to form stream segregation, which can be facilitated by voluntary attention.

### Conflict of interest statement

The authors declare that the research was conducted in the absence of any commercial or financial relationships that could be construed as a potential conflict of interest.

## Footnotes

^1^It would be of interest to learn the amount of time needed for training to reach a stable performance level prior to the neurophysiological experiment using the current stimulus paradigm. However, this experiment and the behavioral study prior to it did not provide sufficient data to address this question. The subjects in the previous behavioral study had completed the experiments before the question of training arose, and thus this project did not address the training effect over time.
